# Expanding the Orthologous Matrix (OMA) programmatic interfaces: REST API and the *OmaDB* packages for R and Python

**DOI:** 10.12688/f1000research.17548.2

**Published:** 2019-03-29

**Authors:** Klara Kaleb, Alex Warwick Vesztrocy, Adrian Altenhoff, Christophe Dessimoz

**Affiliations:** 1 Centre for Life’s Origins and Evolution, Department of Genetics, Evolution and Environment, University College London, London, WC1E 6BT, UK; 2 Swiss Institute of Bioinformatics, Lausanne, Switzerland; 3 Department of Computer Science, ETH Zurich, Zurich, Switzerland; 4 Department of Computer Science, University College London, London, WC1E 6BT, UK; 5 Department of Computational Biology, University of Lausanne, Lausanne, 1015, Switzerland; 6 Center for Integrative Genomics, University of Lausanne, Lausanne, 1015, Switzerland

**Keywords:** orthologs, paralogs, hierarchical orthologous groups, comparative genomics, orthologous matrix, oma, API, R, python, REST, bioconductor

## Abstract

The Orthologous Matrix (OMA) is a well-established resource to identify orthologs among many genomes. Here, we present two recent additions to its programmatic interface, namely a REST API, and user-friendly R and Python packages called *OmaDB*. These should further facilitate the incorporation of OMA data into computational scripts and pipelines. The REST API can be freely accessed at https://omabrowser.org/api. The R OmaDB package is available as part of Bioconductor at http://bioconductor.org/packages/OmaDB/, and the omadb Python package is available from the Python Package Index (PyPI) at https://pypi.org/project/omadb/.

## Introduction

Orthologs are pairs of protein coding genes that have common ancestry and have diverged due to speciation events ^1^. The detection of orthologs is of fundamental importance in many fields in biology, such as comparative genomics, as it allows us to propagate existing biological knowledge to ever growing newly sequenced data ^2, 3^.

The Orthologous Matrix (OMA) is a method and resource for the inference of orthologs among complete genomes ^4^. The OMA database ( https://omabrowser.org) features broad scope and size with currently over 2,100 species from all three domains of life.

The OMA browser has supported multiple ways of exporting the underlying data from its beginning. Users can download data either via bulk archives or interactively through the browser—using where possible standard file formats, such as FASTA, OrthoXML ^5^, or PhyloXML ^6^. For programmatic access, early OMA database releases offered an Application Programming Interface (API) in the form of the Simple Object Access Protocol (SOAP). However, the complexity and limited adoption of SOAP has prompted us to recently switch to the simpler, faster, and more widely used Representational State Transfer (REST) protocol for the OMA API ^4^. Here, we provide a description of this new OMA REST API.

Furthermore, the R environment is widely used in bioinformatics due to its flexibility as a high-level scripting language, statistical capabilities, and numerous bioinformatics libraries. In particular, the Bioconductor open source framework contains over 2,000 packages to facilitate either access to or manipulation of biological data ^7^. This motivated us to develop the OmaDB Bioconductor package which provides a more idiomatic and user-friendly access to OMA data in R implemented on top of the REST API.

Finally, to also enable Python users to easily interact with the database, we have developed a similar package in that language, compliant with the conventions and with support of typical complementary Python packages as outlined below.

## Methods

We start by describing the OMA REST API, before moving on to detail the OmaDB Bioconductor package, and finally outline the omadb Python package.

### OMA REST API

The REST framework is an API architectural style that is based on URLs and HTTP protocol methods. It was designed to be stateless and thus is context independent. That is, it does not save data internally between the HTTP requests which minimises server-side application state, thus easing parallelism. The combination of the HTTP and JSON data formats makes it particularly suitable for web applications and easily supported by most programming languages.

Since the backend of the OMA browser is almost fully based on Python and its frontend is supported by the Django web framework ^8^, we have opted to use the Django Rest Framework (DRF) to implement a REST API in our latest release ^4^. Most API calls require querying the OMA database, stored in HDF5 ^9^, using a custom Python library (“pyoma”). The query results are serialised in the format requested by the user — typically JSON.

**Figure 1.**
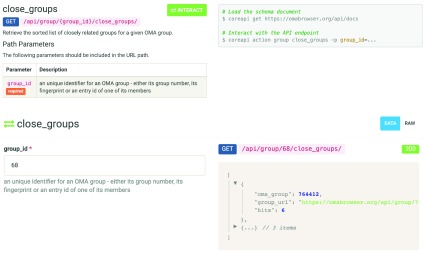
Showcase of the OMA REST API documentation page, with an example of the interactive query and response.

Most data available through the OMA browser is now also accessible via the API, with the exception of the local synteny data. This includes individual genes and their attributes such as protein or cDNA sequences, cross-references, pairwise orthologs, hierarchical orthologous groups ^10^, as well as species trees and the corresponding taxonomy. The API documentation as well as the interactive interface can be found at https://omabrowser.org/api/docs ( Figure 1).

### OmaDB Bioconductor package

To facilitate simplified access to the API and downstream analyses in the R environment, we have also developed an API wrapper package in R, now available in Bioconductor ^7^ ( http://bioconductor.org/packages/OmaDB/). This allowed for abstraction of the server interface, eliminating the need to know structure of the database or the URL endpoints to access the required data.

The package consists of a collection of functions that import OMA data into R objects, the type of which depends on the query supplied. Due to the volume of the data available, some selected object attributes are at first given as URL endpoints. However, these are automatically loaded upon accession. OmaDB also facilitates further downstream analyses with other Bioconductor packages, such as GO enrichment analysis with topGO ^11^, sequence analysis with BioStrings ^12^, phylogenetic analyses using ggtree ^13^ or gene locus analyses with the help of GenomicRanges ^14^.

The open source code is hosted at https://github.com/DessimozLab/OmaDB/. In the results section we showcase usage of the latest version of the package (v2.0), which requires R version >= 3.6 and Bioconductor version >= 3.9. Note that as of the time of publication, this is in the Bioconductor development version. For details, see the Software Availability section.

***Package Installation***


if (!requireNamespace("BiocManager"))
install.packages("BiocManager")
BiocManager::install("OmaDB")
# Load the package
library(OmaDB)


### omadb Python package

For Python users, we provide an analogous package named *omadb*. Results are supplied to users as a hybrid attribute-dictionary object. As such, both attribute and key-based access is possible. Where the URL of a further API call is listed in a response, this has been designed to be automatically requested for the user.

For data that can be represented as a table, the *pandas* package ^15^ is supported. HOGs can be analysed or displayed using the *pyham* library ^16^. Trees are retrievable as *DendroPy* ^17^ or *ETE3* ^18^ Tree objects. Gene Ontology enrichment analyses are possible through the use of the *goatools* package ^19^.

 The open source code is hosted at https://github.com/DessimozLab/pyomadb/. The package requires Python >=3.6, as well as a stable internet connection. It is also available to download from PyPI, installable using pip.

***Package Installation***


# Install in shell, using pip
$ pip install omadb

# In Python, load the package
>>>fromomadb import Client
# Initialise the client
>>> c = Client()


## Results

We provide six illustrative examples in R. The first shows a direct call to the REST API, while the other five showcase the OmaDB R package (version 2.0). These examples are also available as a Jupyter notebook ^20^ as part of the OmaDB R code repository. We have also provided analogous examples in Python, also in the form of a Jupyter notebook, included in its code repository—with the exception of Example 6, which uses a package only available in R.

Note that the results of the queries using the API and the packages may change as we continue to update the OMA database. The OMA database release of June 2018 was used to generate the examples below. 

### Example 1 - Simply accessing the API, in R, via URLs

One way to access the API is to directly send a request using httr ^21^ in R. This approach requires the user to know the URL of the API endpoint, as well as the URL of the API function of interest. Some additional processing steps of the resultant response is usually needed. A simple example to retrieve information on the P53_RAT protein is provided below.

Here we first formulate our URL of interest and use it to send a GET request to the API. This gives us the response JSON object, which can then be parsed into an R list.


library(httr)

url <-"https://omabrowser.org/api/protein/P53_RAT/"
response <- GET(url)

response_content_list <- httr::content(response, as ="parsed")


### Example 2 - Using a sequence to find its gene family (Hierarchical Orthologous Group) and function via gene ontologies

Below is a simple workflow using the OmaDB package to annotate a given protein sequence, using the mapSequence() function.


library(OmaDB)

sequence <-'MKLVFLVLLFLGALGLCLAGRRRSVQWCAVSQPEATKCFQWQRNMRKVRGPPVSCIKRD
SPIQCIQAIAENRADAVTLDGGFIYEAGLAPYKLRPVAAEVYGTERQPRTHYYAVAVVKKGGSFQLNELQGL
KSCHTGLRRTAGWNVPIGTLRPFLNWTGPPEPIEAAVARFFSASCVPGADKGQFPNLCRLCAGTGENKCAFS
SQEPYFSYSGAFKCLRDGAGDVAFIRESTVFEDLSDEAERDEYELLCPDNTRKPVDKFKDCHLARVPSHAVV
ARSVNGKEDAIWNLLRQAQEKFGKDKSPKFQLFGSPSGQKDLLFKDSAIGFSRVPPRIDSGLYLGSGYFTAI
QNLRKSEEEVAARRARVVWCAVGEQELRKCNQWSGLSEGSVTCSSASTTEDCIALVLKGEADAMSLDGGYVY
TAGKCGLVPVLAENYKSQQSSDPDPNCVDRPVEGYLAVAVVRRSDTSLTWNSVKGKKSCHTAVDRTAGWNIP
MGLLFNQTGSCKFDEYFSQSCAPGSDPRSNLCALCIGDEQGENKCVPNSNERYYGYTGAFRCLAENAGDVAF
VKDVTVLQNTDGNNNEAWAKDLKLADFALLCLDGKRKPVTEARSCHLAMAPNHAVVSRMDKVERLKQVLLHQ
QAKFGRNGSDCPDKFCLFQSETKNLLFNDNTECLARLHGKTTYEKYLGPQYVAGITNLKKCSTSPLLEACEF
LRK'

seq_annotation <- mapSequence(sequence)
length(seq_annotation$targets)*# 1*


The identified targets can be found in the seq_annotation$targets. As the length of this object attribute is 1, in this example the sequence mapping identified a single target sequence. From this object further information can be obtained as follows:


seq_annotation$targets[[1]]$canonicalid# 'TRFL_HUMAN'


Thus, our sequence is human lactotransferrin (also known as lactoferrin). Lactotransferrin is one of four subfamilies of transferrins in mammals ^22^.

To investigate the evolutionary history of genes more precisely, we turn to Hierarchical Orthologous Groups (HOGs)—sets of genes which have descended from a single common ancestral gene within a taxonomic range of interest ^10^. For an introduction to HOGs, we refer the interested reader to the following short video: https://youtu.be/5p5x5gxzhZA.

By knowing the ID of the HOG to which our sequence belongs, we can obtain a list of all the HOG members (i.e. all genes in the HOG), as follows:


hog_id <- seq_annotation$targets[[1]]$oma_hog_id# 'HOG:0413862.1a.1b'
hog <- getHOG(id = hog_id, members = TRUE, level ='Mammalia')
hog$members


Note that it is also possible to access information on a HOG using the getHOG() function. A HOG can be identified by its ID or the ID of one of its member proteins. Therefore the below will produce the same output.


hog <- getHOG(id ='TRFL_HUMAN', members = TRUE, level ='Mammalia')


We can easily retrieve the Gene Ontology (GO) terms ^23^ that are associated to each of the members using OmaDB.


go_annotations <- getProtein(hog$members$omaid,attribute ='gene_ontology')


The resultant list of GO terms per gene is in the “geneID2GO” format by default, which is used by the topGO ^11^ package.

To compare the function of lactotransferrins with their paralogous counterparts, we can retrieve a background set consisting of all members of the transferring HOG defined at the root of the eukaryotes


bgHOG <- getHOG(id ='TRFL_HUMAN', members = TRUE, level ='Eukaryota')
bgAnnnot <- getProtein(bgHOG$members$omaid, attribute ='gene_ontology')


We can now construct a topGO object using the getTopGO function as seen below. Note that the background set of terms is set by getTopGO to all terms appearing in the list of annotations. This may not be appropriate in all cases—the choice of background set requires careful consideration ^24^.


bgAnnnotFormatted = formatTopGO(bgAnnnot, format ='geneID2GO')

library(topGO)
myGO <- getTopGO(annotations = bgAnnnotFormatted, format ='geneID2GO',foregroundGenes = hog$members$entry_nr, ontology ='BP')

myRes <- runTest(myGO, algorithm ='classic', statistic ='fisher')
print(GenTable(myGO, myRes))


As the output in Table 1 indicates, several enriched terms in the mammalian lactotransferrin are related to bone formation, consistent with previous reports in the literature (e.g. 25). So is the role of lactotransferrin in antimicrobial activity (e.g. 26).

**Table 1.** Gene Ontology enrichment of Biological Process terms associated with mammalian lactotransferrins compared to all eukaryotic transferrins, as obtained from example 2.

GO.ID	Term	P-value
GO:0001501	skeletal system development	<1e-30
GO:0001503	ossification	<1e-30
GO:0001649	osteoblast differentiation	<1e-30
GO:0001816	cytokine production	<1e-30
GO:0001817	regulation of cytokine production	<1e-30
GO:0001818	negative regulation of cytokine production	<1e-30
GO:0002237	response to molecule of bacterial origin	<1e-30
GO:0002682	regulation of immune system process	<1e-30
GO:0002683	negative regulation of immune system process	<1e-30
GO:0002761	regulation of myeloid leukocyte differentiation	<1e-30

### Example 3 - Taxonomic tree visualisation

The taxonomic data obtained using the OmaDB package can easily be plugged into ggtree ^13^ for phylogenetic tree visualisation. First, the tree is obtained using the getTaxonomy() function. In this example, the tree is rooted at the Hominoidea taxonomic level. The default format of the object returned is newick.


tax <- getTaxonomy(root ='Hominoidea')


The resultant object can directly be used to build a phylogenetic tree using the ggtree package as below:


library(ggtree)
tree <- getTree(tax$newick)

mytree <- ggtree(tree)


**Figure 2.**
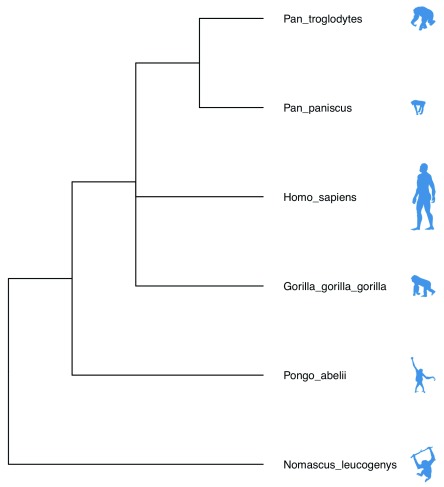
Species taxonomy tree obtained using example 3.

The tree can be further annotated using species silhouettes from PhyloPic ( http://phylopic.org/). This functionality is already enabled within the ggtree package and just requires obtaining the relevant image codes. The workflow to produce Figure 2 is below.


library(rphylopic)

labels <- tree$tip.label

labelsFormatted <- sapply(labels, FUN = function(x)
           gsub("_"," ", x, fixed = TRUE))

ids <- sapply(labelsFormatted, FUN = function(x)         
           name_search(x)$canonicalName[1,1])

images <- sapply( as.character(ids), FUN = function(x)  
           tryCatch(name_images(x)$same[[1]]$uid, error = 
           function(w) name_images(x)$supertaxa[[1]]$uid) )

d <- data.frame(label = labels, images = as.character(images))

library(dplyr)
library(ggimage)



mytree %<+% d + geom_tiplab(aes(image = images), geom ='phylopic', 
    offset = 2.3, color ='steelblue') + geom_tiplab(offset = 0.3)    
    + ggplot2::xlim(0, 7)


### Example 4 - Visualising the distribution of PAM distances in the taxonomic space

To obtain all orthologous pairs between two genomes, we can use the getGenomePairs() function. To limit server load, the resultant response is paginated and by default only returns the first page, capped at 100 entries. This is easily adjustable by setting the ‘per_page’ parameter to either the number of orthologs required or simply to ‘all’.

In this example, we compare the distribution of PAM distances (Point accepted mutations; 27) between orthologs of two species-pairs, namely human-dog and human-mouse. First, we request the required data:


mouse_id = getGenome(id='Mus musculus')$taxon_id
human_id = getGenome(id='Homo sapiens')$taxon_id
dog_id = getGenome(id='Canis lupus familiaris')$taxon_id

human_mouse <- getGenomePairs(genome_id1 = human_id, 
    genome_id2 = mouse_id, rel_type ='1:1')

human_dog <- getGenomePairs(genome_id1 = human_id, 
    genome_id2 = dog_id, rel_type ='1:1')


**Figure 3.**
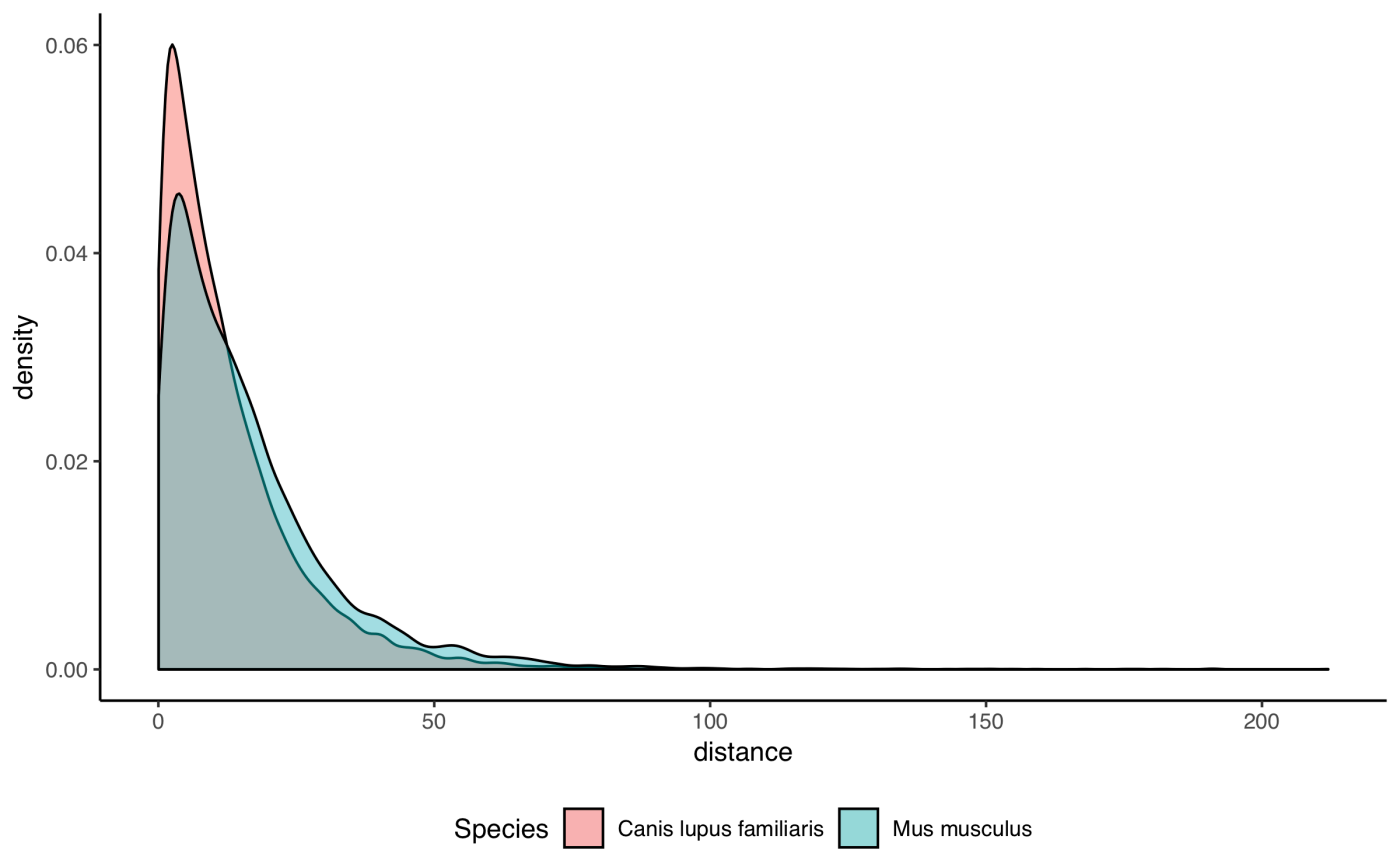
Distribution of evolutionary distances (in PAM units; 27) human-dog (red) and human-mouse (blue) pairs, obtained using example 4.

We can then bind the two resultant data frames and plot the results ( Figure 3), as so:


human_mouse$Species <-'Mus musculus'
human_dog$Species <-'Canis lupus familiaris'

all_pairs <- rbind(human_mouse, human_dog)
all_pairs$Species <- as.factor(all_pairs$Species)

library(ggplot2)

g <- ggplot(all_pairs, aes(x =distance, fill = Species)) +
    geom_density(alpha = 0.5) + 
    xlab('evolutionary distance [PAM]') +
    theme(legend.position ='bottom', panel.grid.major = 
    element_blank(), panel.grid.minor = element_blank(),
    panel.background = element_blank(), axis.line = element_line(colour 
    ='black'))
print(g)


The two-sample Kolmogorov-Smirnov test can be performed on the two distributions, using the command:


ks.test(human_dog$distance, human_mouse$distance)


This returns p-value < 2.2e-16. The median distance between dog and human is shorter than that of mouse and human (8.8 *vs.* 11.8). This is consistent with previous observations that the rodent has a longer branch than humans and carnivores, in part due to their shorter generation time ^28^.

### Example 5 - Annotating protein sequences not present in OMA

Although the OMA database currently analyses over 2,100 genomes, many more have been sequenced, and the gap keeps on widening. It is nevertheless possible to use OMA to infer the function of custom protein sequences through a fast approximate search against all sequences in OMA ^4^.


# Our mystery sequence is cystic fibrosis transmembrane conductance
# regulator in the Emperor penguin (UniProt ID: A0A087RGQ1_APTFO)
mysterySeq <-
'FFFLLRWTKPILRKGYRRRLELSDIYQIPSADSADNLSEKLEREWDRELATSKKKPKLINALRRCFFWKFM
FYGIILYLGEVTKSVQPLLLGRIIASYDPDNSDERSIAYYLAIGLCLLFLVRTLLIHPAIFGLHHIGMQMRI
AMFSLIYKKILKLSSRVLDKISTGQLVSLLSNNLNKFDEGLALAHFVWIAPLQVALLMGLLWDMLEASAFSG
LAFLIVLAFFQAWLGQRMMKYRNKRAGKINERLVITSEIIENIQSVKAYCWEDAMEKMIESIRETELKLTRK
AAYVRYFNSSAFFFSGFFVVFLAVLPYAVIKGIILRKIFTTISFCIVLRMTVTRQFPGSVQTWYDSIGAINK
IQDFLLKKEYKSLEYNLTTTGVELDKVTAFWDEGIGELFVKANQENNNSKAPSTDNNLFFSNFPLHASPVLQ
DINFKIEKGQLLAVSGSTGAGKTSLLMLIMGELEPSQGRLKHSGRISFSPQVSWIMPGTIKENIIFGVSYDE
YRYKSVIKACQLEEDISKFPDKDYTVLGDGGIILSGGQRARISLARAVYKDADLYLLDSPFGHLDIFTEKEI
FESCVCKLMANKTRILVTSKLEHLKIADKILILHEGSCYFYGTFSELQGQRPDFSSELMGFDSFDQFSAERR
NSILTETLRRFSIEGEGTGSRNEIKKQSFKQTSDFNDKRKNSIIINPLNASRKFSVVQRNGMQVNGIEDGHN
DPPERRFSLVPDLEQGDVGLLRSSMLNTDHILQGRRRQSVLNLMTGTSVNYGPNFSKKGSTTFRKMSMVPQT
NLSSEIDIYTRRLSRDSVLDITDEINEEDLKECFTDDAESMGTVTTWNTYFRYVTIHKNLIFVLILCVTVFL
VEVAASLAGLWFLKQTALKANTTQSENSTSDKPPVIVTVTSSYYIIYIYVGVADTLLAMGIFRGLPLVHTLI
TVSKTLHQKMVHAVLHAPMSTFNSWKAGGMLNRFSKDTAVLDDLLPLTVFDFIQLILIVIGAITVVSILQPY
IFLASVPVIAAFILLRAYFLHTSQQLKQLESEARSPIFTHLVTSLKGLWTLRAFGRQPYFETLFHKALNLHT
ANWFLYLSTLRWFQMRIEMIFVVFFVAVAFISIVTTGDGSGKVGIILTLAMNIMGTLQWAVNSSIDVDSLMR
SVGRIFKFIDMPTEEMKNIKPHKNNQFSDALVIENRHAKEEKNWPSGGQMTVKDLTAKYSEGGAAVLENISF
SISSGQRVGLLGRTGSGKSTLLFAFLRLLNTEGDIQIDGVSWSTVSVQQWRKAFGVIPQKVFIFSGTFRMNL
DPYGQWNDEEIWKVAEEVGLKSVIEQFPGQLDFVLVDGGCVLSHGHKQLMCLARSVLSKAKILLLDEPSAHL
DPVTSQVIRKTLKHAFANCTVILSEHRLEAMLECQRFLVIEDNKLRQYESIQKLLNEKSSFRQAISHADRLK
LLPVHHRNSSKRKPRPKITALQEETEEEVQETRL'
myAnnotations <- annotateSequence(mysterySeq)


This results in 54 GO annotations. By comparison, this sequence has merely 15 GO annotations in UniProt-GOA ^29^ — all of which are also predicted by this method in OMA.

### Example 6 - Combining OmaDB with BgeeDB for gene expression

We go back to the lactotransferrin gene family from Example 2. We can use OmaDB in conjunction with the BgeeDB Bioconductor package ^30^ to retrieve expression data from the Bgee database ^31^ as follows.


BiocManager::install("BgeeDB")
library(BgeeDB)

# Bgee uses Ensembl gene IDs, obtainable using OmaDB’s cross-references.
trfl_xrefs <- getProtein(id='TRFL_HUMAN')$xref
trfl_ens_id <- subset(trfl_xrefs, source =='Ensembl Gene')$xref
# The Ensembl gene IDs need to be without version suffix
trfl_ens_id <- strsplit(trfl_ens_id,'.',fixed=TRUE)[[1]][1]

my_stage <-'UBERON:0034920'# Infant stage
bgee.expr <- Bgee$new(species='Homo_sapiens')
expr.data <- loadTopAnatData(bgee.expr, stage = my_stage)
gene.expr.tissue.ids <- 
    unlist(expr.data$gene2anatomy[trfl_ens_id], use.names = F)
tissues <- expr.data$organ.names
print(tissues[tissues$ID %in% gene.expr.tissue.ids, ])



Among the tissues in which lactotransferrin is expressed according to Bgee ( Table 2), we note the bone marrow and the palpebral conjunctiva (the eyelid inner surface). This is consistent with the aforementioned involvement of lactotransferrin in bone formation and anti-microbial activity.

**Table 2.** Human tissues in which lactotransferrin is expressed in infant stage, according to the Bgee database version 14 (output of Example 6).

ID	Name
UBERON:0001812	palpebral conjunctiva
UBERON:0000178	blood
UBERON:0002371	bone marrow
UBERON:0001154	vermiform appendix
UBERON:0002084	heart left ventricle

Further tutorials on the OmaDB package can be found in the accompanying vignettes:


browseVignettes('OmaDB')


## Discussion and outlook

Orthology is used for various purposes, such as species tree inference, gene evolution dynamic, or protein function prediction. The retrieval of orthologs is thus typically just the starting point of a larger analysis. Therefore, this overhaul and expansion of the OMA programmatic interface will facilitate the incorporation of OMA data in such larger analyses.

Our R package will continue to be maintained in line with the biannual Bioconductor releases. Further work to improve the package includes improvement in performance. For example, the responses are currently fully loaded into an R object of choice which, depending on the response size, may create some time lag in the response. We will also continue to update the package and the API in sync with the OMA browser to incorporate new functionalities of OMA.

Likewise, we will also maintain and further develop the Python package. In particular, we will explore the possibility of further integration with the BioPython library ^32^.

More generally, in OMA we will keep supporting the various ways of accessing the underlying data, including the interactive web browser and flat files in a variety of formats. The REST API is also complemented by a new SPARQL interface that enables highly specific queries, as well as federated queries over multiple resources ^4^. However, the query language is more complex.

We very much welcome feedback and questions from the community. We also highly appreciate contributions to the code in the form of pull requests. Our preferred channel for support is the BioStar website ^33^, where we monitor all posts with keyword “oma”.

## Software availability

Please note that this manuscript uses version 2.0 of the OmaDB R package, which is in the **development version** of Bioconductor (v.3.9). Until the release of Bioconductor v.3.9 in Spring 2019, there are two possible ways of installing it:

1) Install the development version of R (v.3.6) — required for Bioconductor v.3.9 — and install OmaDB using the command:
BiocManager::install('OmaDB', version ='devel')
–or–
2) Install OmaDB 2.0 directly from the github repo using the devtools R package:
install.packages('devtools')
library(devtools)
install_github('dessimozlab/omadb')

REST API available from: https://omabrowser.org/api

Documentation available from: https://omabrowser.org/api/docs

R OmaDB package available from: http://bioconductor.org/packages/OmaDB/

Source code available from: https://github.com/DessimozLab/OmaDB/

Archived source code as at time of publication: http://doi.org/10.5281/zenodo.2595086 ^34^

License: GPL-2

omadb Python package available from: https://pypi.org/project/omadb/

Source code available from: https://github.com/DessimozLab/pyomadb/

Archived source code as at time of publication: http://doi.org/10.5281/zenodo.2530250 ^35^

License: GPL-3

We also provide a binder to reproduce in Python the analyses done in R. This is available from: https://mybinder.org/v2/gh/DessimozLab/pyomadb/master?filepath=examples%2Fpyomadb-examples.ipynb
