# Supplementary material for: Expanding the Orthologous Matrix (OMA) programmatic interfaces: REST API and the OmaDB packages for R and Python
Source: F1000Res. 2019 Mar 29;8:42. Originally published 2019 Jan 10. [Version 2] doi: 10.12688/f1000research.17548.2 (PMC6464060; doi:10.12688/f1000research.17548.2)
Supplement: Peer review history

# Peer review: Reviewer response for version 2

**DOI:** 10.5256/f1000research.20395.r46493

**Published:** 2019-04-12

**Authors:** Laurent Gatto

**Affiliations:** 1 University of Louvain (UCLouvain), Brussels, Belgium

Thank you very much to the authors for their detailed reply, carefully addressing all my points and suggestions.

I confirm that I have read this submission and believe that I have an appropriate level of expertise to confirm that it is of an acceptable scientific standard.

# Peer review: Reviewer response for version 2

**DOI:** 10.5256/f1000research.20395.r46494

**Published:** 2019-04-01

**Authors:** Bastian Greshake Tzovaras, Ngoc-Vinh Tran

**Affiliations:** 1 Environmental Genomics and Systems Biology Division, Lawrence Berkeley National Laboratory (LBNL), Berkeley, CA, USA; 2 Department for Applied Bioinformatics, Institute of Cell Biology and Neuroscience, Goethe University, Frankfurt am Main, Germany

The authors have successfully addressed all prior comments.

We confirm that we have read this submission and believe that we have an appropriate level of expertise to confirm that it is of an acceptable scientific standard.

# Peer review: Reviewer response for version 1

**DOI:** 10.5256/f1000research.19190.r42908

**Published:** 2019-02-11

**Authors:** Laurent Gatto

**Affiliations:** 1 University of Louvain (UCLouvain), Brussels, Belgium

Kaleb et al. present two packages, namely R/Bioconductor OmoDB and python omadb, that allow users to query and use data from the Orthologous Matrix database. The article is well written and the authors provide 6 examples that convincingly demonstrate the usefulness and reach of their work.

 I have a couple of comments and suggestions below, presented in chronological order. The only serious one is a request for the authors to describe the outputs in their examples a bit more (see below for details), to facilitate the adoption for users that wouldn't be familiar with R.

- In https://omabrowser.org/api/docs, the pagination example has a typo. The genomes should be replaced with genome:

```

 $ "https://omabrowser.org/api/genomes/?page=2"

 HTTP/1.1 404 Not Found

 Server: nginx

 Date: Mon, 11 Feb 2019 06:04:30 GMT

 Content-Type: text/html; charset=utf-8

 Connection: keep-alive

 X-Frame-Options: SAMEORIGIN

 Vary: Cookie

 Set-Cookie: __utmmobile=d41d8cd98f00b204e9800998ecf8427e; expires=Wed, 10-Feb-2021 06:04:30 UTC; Path=/

 Set-Cookie: sessionid=9zb42ljib7apkubml1e1t742i5p6f3a6; expires=Mon, 25-Feb-2019 06:04:30 GMT; HttpOnly; Max-Age=1209600; Path=/

 $ curl -I "https://omabrowser.org/api/genome/?page=2"

 HTTP/1.1 200 OK

 Server: nginx

 Date: Mon, 11 Feb 2019 06:04:32 GMT

 Content-Type: application/json

 Connection: keep-alive

 Link: ; rel="first", ; rel="prev", ; rel="next", ; rel="last"

 X-Total-Count: 2198

 Vary: Accept, Cookie

 Allow: GET, HEAD, OPTIONS

 X-Frame-Options: SAMEORIGIN

 Set-Cookie: __utmmobile=d41d8cd98f00b204e9800998ecf8427e; expires=Wed, 10-Feb-2021 06:04:32 UTC; Path=/

 Set-Cookie: sessionid=9h5n3ouuwvh4ock9dz3q4yiprmb8iw0d; expires=Mon, 25-Feb-2019 06:04:32 GMT; HttpOnly; Max-Age=1209600; Path=/

 Strict-Transport-Security: max-age=15768000

 ```

- In the introduction, the authors explain that 'Most data available through the OMA browser is now accessible via the API'. I think it would be useful to know what data isn't available and whether the browser and REST API would ever be equivalent in terms of data served. This might be partly addressed later, in the discussion, where the authors mention 'support for local synteny'. Some additional details would be useful to redirect users to the appropriate interface. Similarly, it would be useful to know if the R and python packages provide access to the same data, or if differences also exist there.
- I didn't see mention of the R and python packages on the OmaDB web page. This would be a useful addition for visitors.
- In the Bioconductor package section, the authors explain that data is provided in 'R friendly objects, namely S3 objects and data frames'. I would suggest to rephrase this and only refer to objects, as S4 objects are also returned and the nature of the technical class system is probably not necessary in the frame of this document.
- Regarding the R package, I would suggest to add URL and BugReports fields in the packages DESCRIPTION file. This helps users find the GitHub repository and report issues. I also noted that in the 'getting started' vignette, it looks like some section a missing a space after the section markup. I have send a pull request fixing these and some other minor issue.
- Note that the html and R version of the vignette shouldn't be included in the package source.
- In the python package section, the authors mention that this package is *also* named 'omadb'. I would argue that the packages have different names, as programming languages are case sensitive and suggest to drop the also to avoid any confusion.
- In the first sentence of the result section, authors should replace R library by R package, as they are referring to their package, not the location where the package is being installed (the library).
- In general, it would be very useful for the authors to describe the different outputs they have. I am not expecting the authors to provide full details of the REST API responses, but describing how the results match the text would be important. For example, in example 1, they only show how to produce the `response_content_list` response. Here, it would be useful to explain that this R list directly maps the REST json message, and point to the specific documentation entry point. Such an explanation motivates the example in the text and helps users, that aren't familiar with REST, to understand the relation between the server and the package.
- Similarly in example 2, the authors create the `seq_annotation` variable and mention that only one target sequence was identified. Here, it would be useful to show that `length(seq_annotation$targets)` is equal to 1, to back their claim, to indicate how users can verify the number of targets, and motivate the use of the first list index in later code chunks.
- Still in example 2, the authors query and extract the hog members. These data are however already present in the first output of that example, under `seq_annotation$targets[[1]]$oma_hog_members`. It would be useful to explain why the authors send a second query to obtain that data and clarify whether `oma_hog_members` is always equivalent to calling `getHOG` and `getProtein`.
- When trying to reproduce the code, I first failed to run the code chunks calling `getProtein`. Later, the authors clarify the software requirements in more details. It would however be useful to briefly mention, early on in the Results section, what version was used for the examples.
- In example 5, I would suggest to update to new function name, as `getAnnotations` is expected to be deprecated in the next release, especially as the new version of the package is anyway required for the `getProtein` function.

```

 > myAnnotations <- getAnnotation(mysterySeq)

 Warning message:

 'getAnnotation' is deprecated.

 Use 'annotateSequence' instead.

 See help("Deprecated")

 ```

- Another example where an explanation of the output is important is example 5. The authors call `myAnnotation <- getAnnotations(mysterySeq)` and then refer to 54 GO annotation results. In repeating their analysis, I obtain a data frame with 55 observations (see below). It is this unclear whether I have a different result, if one observation should be dropped, or if my output is completely wrong (was I even expecting a data frame?).

```

 > dim(myAnnotations)

 [1] 55 13

 ```

- In general, given the nature of the package, i.e. that it accesses an online repository that is (or can be) updated regularly, results may change, this also explaining why I may have different results.

I confirm that I have read this submission and believe that I have an appropriate level of expertise to confirm that it is of an acceptable scientific standard, however I have significant reservations, as outlined above.

# Response

**Published:** 2019-03-19

**Authors:** Christophe Dessimoz

**Affiliations:** Computational Evolutionary Biology and Genomics, University of Lausanne, Switzerland

*In https://omabrowser.org/api/docs, the pagination example has a typo. The genomes should be replaced with* *genome* *.*

 RESPONSE: Thanks for spotting this typo. It has been fixed.

*In the introduction, the authors explain that 'Most data available through the OMA browser is now accessible via the API'. I think it would be useful to know what data isn't available and whether the browser and REST API would ever be equivalent in terms of data served. This might be partly addressed later, in the discussion, where the authors mention 'support for local synteny'. Some additional details would be useful to redirect users to the appropriate interface. Similarly, it would be useful to know if the R and python packages provide access to the same data, or if differences also exist there.*

 RESPONSE: As mentioned in the Discussion, the package currently lacks availability of the data on local synteny, mainly due to the complexity of the data representation in the API format.  We aim to bridge this gap in the next release. We have now amended the Methods section to reflect the difference between OMA browser and the API more explicitly, as well as the discussion to reassure the users the API and OMA browser will be kept in sync with further OMA developments.

 Both R and Python packages use the same API and supply the same data.

*I didn't see mention of the R and python packages on the OmaDB web page. This would be a useful addition for visitors.*

 RESPONSE: We already had a link to the R package in the /api/docs, but we have now also included link directly from the "compute" menu.

*In the Bioconductor package section, the authors explain that data is provided in 'R friendly objects, namely S3 objects and data frames'. I would suggest to rephrase this and only refer to objects, as S4 objects are also returned and the nature of the technical class system is probably not necessary in the frame of this document.*

 RESPONSE: This has now been amended.

*Regarding the R package, I would suggest* *to add* *URL and BugReports fields in the packages DESCRIPTION file. This helps users find the GitHub repository and report issues. I also noted that in the 'getting started' vignette, it looks like some section a missing* *a space* *after the section markup. I have* *send* *a pull request fixing these and some other minor issue.*

 RESPONSE: Thank you for the pull request, this has now been merged with OmaDB  version 2.0.

*Note that the* *html* *and R version of the vignette shouldn't be included in the package source.*

 RESPONSE: This has now been amended.

*In the python package section, the authors mention that this package is also named '* *omadb* *'. I would argue that the packages have different names, as programming languages are case sensitive and suggest to drop the also to avoid any confusion.*

 RESPONSE: Amended as requested.

*In the first sentence of the result section, authors should replace R library by R package, as they are referring to their package, not the location where the package is being installed (the library).*

 RESPONSE: This has now been amended.

*In general, it would be very useful for the authors to describe the different outputs they have. I am not expecting the authors to provide full details of the REST API responses, but describing how the results match the text would be important. For example, in example 1, they only show how to produce the `response_content_list` response. Here, it would be useful to explain that this R list directly maps the REST* *json* *message, and point to the specific documentation entry point. Such an explanation motivates the example in the text and helps users, that aren't familiar with REST, to understand the relation between the server and the package.*

 RESPONSE: We agree, and further information on the output generated in the manuscript has now been added to example 1.

*Similarly* *in example 2, the authors create the `seq_annotation` variable and mention that only one target sequence was identified. Here, it would be useful to show that `length(seq_annotation$targets)` is equal to 1, to back their claim, to indicate how users can verify the number of targets, and motivate the use of the first list index in later code chunks.*

 RESPONSE: This has now been updated.

*Still* *in example 2, the authors query and extract the hog members. These data are however already present in the first output of that example, under `seq_annotation$targets[[1]]$oma_hog_members`. It would be useful to explain why the authors send a second query to obtain that data and clarify whether `oma_hog_members` is always equivalent to calling `getHOG` and `getProtein`.*

 RESPONSE: It is true that the hog members can also be directly accessed via the oma_hog_members attribute. However, the members are only loaded once the attribute is accessed, so we do not add unnecessary requests. Even more importantly, if we would load the hog members via the oma_hog_members attribute, it is not obvious for which taxonomic level the members are loaded. We therefore prefer to keep the current slightly more verbose way to access the data.

*When trying to reproduce the code, I first failed to run the code chunks calling `getProtein`. Later, the authors clarify the software requirements in more details. It would* *however* *be useful to briefly mention, early on in the Results section, what version was used for the examples.*

 RESPONSE: We agree that this can be confusing, and we have now amended the Methods and the Results section to explicitly mention the usage of OmaDB v2.0 for the examples.

*In example 5, I would suggest* *to update* *to* *new* *function name, as `getAnnotations` is expected to be deprecated in the next release, especially as the new version of the package is anyway required for the `getProtein` function.*

 RESPONSE: This was a mistake on our side and has now been amended.

*Another example where an explanation of the output is important is example 5. The authors call `myAnnotation <- getAnnotations(mysterySeq)` and then refer to 54 GO annotation results. In repeating their analysis, I obtain a data frame with 55 observations (see below). It is this unclear whether I have a different result, if one observation should be dropped, or if my output is completely wrong (was I even expecting a data frame?).    In general, given the nature of the package, i.e. that it accesses an online repository that is (or can be) updated regularly, results may change, this also* *explaining* *why I may have different results.*

 RESPONSE: We can confirm that this is due to the December 2018 OMA release, where there are indeed 55 results returned for that particular query. The fact that the results may vary due to the continued updates of the OMA database has now been explicitly mentioned in the beginning of the methods section of the manuscript. We have also added what version of the database was used to generate the examples in the manuscript.

# Peer review: Reviewer response for version 1

**DOI:** 10.5256/f1000research.19190.r42912

**Published:** 2019-02-05

**Authors:** Bastian Greshake Tzovaras, Ngoc-Vinh Tran

**Affiliations:** 1 Environmental Genomics and Systems Biology Division, Lawrence Berkeley National Laboratory (LBNL), Berkeley, CA, USA; 2 Department for Applied Bioinformatics, Institute of Cell Biology and Neuroscience, Goethe University, Frankfurt am Main, Germany

The authors describe a new REST API as an interface to the well-established Orthologous Matrix database. As identifying and evaluating orthologs is a central step in many biological analyses, an easy way to query the over 2,100 species in OMA is highly valuable. To further facilitate querying the data through their API, the authors present packages for R and Python. The API is well documented on the OMA website and the R package comes with vignettes describing different use cases. The manuscript presented here focuses on the OmaDB R-package and showcases some of its functions.

 Being somewhat "ahead of it's time", the R package as described in the manuscript requires both the development version of R (v3.6) and Bioconductor (v3.9). The package installation instructions at the beginning of the manuscript only glances over it, more complete instructions are only found in the *Software availability *section at the end.

 We recommend including more explicit warnings/instructions about the required versions at the beginning, otherwise potential users might be confused when trying to follow along with the examples given in the manuscript (As happened to us and it took us some time to figure out what's going on).

 While the Python package is not extensively discussed in this manuscript, the authors provide a Binder that can be used to reproduce the same analyses using Python. We recommend putting a link to it ( https://mybinder.org/v2/gh/DessimozLab/pyomadb/master?filepath=examples%2Fpyomadb-examples.ipynb) in the manuscript, to help users with taking up the Python library.

 We welcome the switch from the SOAP API to a more modern REST implementation and the provided packages to interface with the API will be valuable for a lot of researchers working with orthologs.

We confirm that we have read this submission and believe that we have an appropriate level of expertise to confirm that it is of an acceptable scientific standard.

# Response

**Published:** 2019-03-19

**Authors:** Christophe Dessimoz

**Affiliations:** Computational Evolutionary Biology and Genomics, University of Lausanne, Switzerland

1. *Being somewhat "ahead of its time", the R package as described in the manuscript requires both the development version of R (v3.6) and Bioconductor (v3.9). The package installation instructions at the beginning of the manuscript only* *glances* *over it, more complete instructions are only found in the Software availability section at the end.*
*We recommend including more explicit warnings/instructions about the required versions at the beginning,* *otherwise* *potential users might be confused when trying to follow along with the examples given in the manuscript (As happened to us and it took us some time to figure out what's going on).*

RESPONSE: We agree that this might cause confusion and we have now updated the OmaDB package section in Methods to mention explicitly that the package version used in the manuscript is 2.0, which until April 2019 is in the development version of Bioconductor. We also point the readers to the Software Availability section where further instructions for package installation are provided. We are hesitant to amend the package installation instructions at the beginning of the manuscript to reflect this as it will change shortly.

1. While the Python package is not extensively discussed in this manuscript, the authors provide a Binder that can be used to reproduce the same analyses using Python. We recommend putting a link to it (https://mybinder.org/v2/gh/DessimozLab/pyomadb/master?filepath=examples%2Fpyomadb-examples.ipynb) in the manuscript, to help users with taking up the Python library.

    RESPONSE: This has now been added.
